# Molecular beam scattering of neon from flat jets of cold salty water†

**DOI:** 10.1039/d5sc01636c

**Published:** 2025-05-30

**Authors:** Walt Yang, Madison M. Foreman, Tiffany C. Ly, Kevin R. Wilson, Daniel M. Neumark

**Affiliations:** a Department of Chemistry, University of California Berkeley CA 94720 USA dneumark@berkeley.edu; b Chemical Sciences Division, Lawrence Berkeley National Laboratory Berkeley CA 94720 USA

## Abstract

Molecular beam scattering experiments are carried out to study collisions between Ne atoms (*E*_i_ = 24.3 kJ mol^−1^) and the surface of a cold salty water (8 m LiBr_(aq)_, 230 K) flat jet. Translational energy distributions are collected as a function of scattering angle using a rotatable mass spectrometer. Impulsive scattering and thermal desorption contribute to the overall scattering distributions, but impulsive scattering dominates at all three incidence angles explored. Highly super-specular scattering is observed in the impulsive scattering channel that is attributed to anisotropic momentum transfer to the liquid surface. The thermal desorption channel exhibits a cos *θ* angular distribution. Compared to Ne scattering from dodecane, fractional energy loss in the impulsive scattering channel is much larger across a wide range of deflection angles. A soft-sphere model is applied to investigate the kinematics of energy transfer between the scatterer and liquid surface. Fitting to this model yields an effective surface mass of 250_−60_^+100^ amu and internal excitation of 11.8 ± 1.6 kJ mol^−1^, both of which are considerably larger than for Ne/dodecane. It thus appears that energy transfer to cold salty water is more efficient than to a dodecane liquid surface, a result attributed to the extensive hydrogen-bonded network of liquid water and roughness of the liquid surface.

## Abstract 2

Molecular beam scattering experiments are carried out to study collisions between Ne atoms (*E*_i_ = 24.3 kJ mol^−1^) and the surface of a cold salty water (8 m LiBr_(aq)_, 230 K) flat jet.

## Graphical abstract

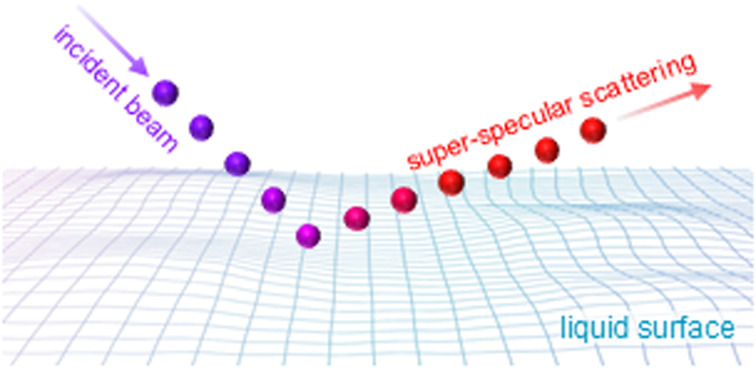

## 1. Introduction

The air–water interface is an important chemical environment for a wide array of chemistries that occur in everyday life. Examples that impact the environment on a global scale include treatment of amine gases,^1^ formation of acid rain,^2–4^ and heterogeneous chemistry on aerosols such as the reactive uptake of N_2_O_5_ that, in turn, modulates ozone concentrations in the atmosphere.^5–9^ These considerations have motivated the development of novel surface-specific experimental methods to study chemical kinetics and dynamics at this unique interface.^10–12^

Several spectroscopic techniques have been designed to characterize the surface region of aqueous solutions.^13–15^ Deep ultraviolet electronic sum frequency generation can probe the presence and photodynamics of ions at the interface through symmetry breaking considerations.^16–24^ Time-resolved second-harmonic generation spectroscopy has uncovered the dynamics of hydrated electrons at the air–water interface,^25^ while vibrational sum frequency generation spectroscopy has been applied to a wide range of problems including charged water structures at the interface,^26–31^ the interfacial assembly of proteins,^32^ and electric double layer dynamics.^33^ Extreme ultraviolet photoelectron spectroscopy has determined interfacial densities of aromatic compounds,^34,35^ while X-ray photoemission spectroscopy has been used to elucidate specific ion effects at aqueous solution–vapor interfaces.^36,37^ Augmented reaction rates compared to those in bulk liquids have been reported through studies on thin liquid films and microdroplets.^38–40^

Molecular beam scattering serves as a complementary method to these techniques and has been incorporated by Nathanson, Minton, McKendrick, Nesbitt, and others into experiments that probe interfacial interactions on a wide variety of liquid surfaces. These experiments were enabled by the development of the wetted wheel by Fenn and Siegbahn as a method to introduce continually-refreshed liquid surfaces into high-vacuum environments.^41,42^ Experiments by Faubel and others on liquid microjets^43–45^ further advanced the field, unlocking the ability to investigate interfacial interactions on more volatile liquids^46^ and thus the vapor–water interface.^47^ Relevant experiments include scattering of HCl, DCl, and N_2_O_5_ from highly concentrated salty water microjets.^48–50^ More complex studies have also been undertaken, including observing solvated electron chemistries at the surface of water using a microjet.^51^ Additionally, surface structures of fluorinated and ionic liquids on a wetted wheel have been deduced through reactive-atom scattering combined with laser-induced fluorescence^52–55^ and rotationally resolved scattering studies.^56,57^

We have recently utilized commercially available microfluidic chips^58^ to create flat jets of volatile liquids in a molecular beam scattering apparatus. Compared to a cylindrical jet, for which the diameter is typically 30 μm, flat jets present a larger scattering target that is more compatible with the dimensions of a molecular beam. Moreover, the well-defined surface normal of a flat jet facilitates the measurement of angular distributions in a scattering experiment.

We have previously performed molecular beam scattering from dodecane flat liquid jets^59–62^ (*P*_vap_ = 1.5 × 10^−2^ Torr at 275 K), in which we reported the angular and translational energy distributions of Ne, CD_4_, ND_3_, and D_2_O evaporating and scattering from dodecane at various scattering geometries.^59–61^ In a recent review article,^62^ we also presented preliminary findings on the evaporation and scattering of Ar from a cold aqueous 8 molal LiBr flat jet (*P*_vap_ = 4.2 × 10^−2^ Torr at 223 K).

For both solvents, the evaporation experiments indicated that the vapor jacket surrounding the jet was sufficiently dilute for scattering experiments to be feasible. Using precedent set by prior gas–solid^63,64^ and gas–liquid^65^ scattering experiments, we characterized two scattering mechanisms at the gas–liquid interface: impulsive scattering (IS) and thermal desorption (TD). Key characteristics for each of these channels are expected in angle-resolved measurements, where IS exhibits near-specular scattering governed by initial conditions while TD yields cos *θ* angular distributions with respect to the surface normal.^63,65–67^

In our dodecane studies, we determined that the physical properties of the scatterers heavily influenced the observed branching ratios between IS and TD. Extracted TD fractions correlated with solubility trends from literature values, and all four scatterers exhibited slightly super-specular scattering behavior in the IS channel. Ne scattering from squalane was previously simulated using classical trajectory calculations,^68^ and our Ne scattering results from dodecane were semiquantitatively produced through molecular dynamics (MD) simulations by Li and co-workers.^69^ From the exploratory results of Ar scattering from cold salty water, the TD channel was favored more than in Ne scattering from dodecane, which may be due to a higher trapping probability at the cold salty water interface, the scatterer identity, or both.

In this work, we extend our capabilities to molecular beam scattering from aqueous solutions and present the first angle-resolved scattering measurements of Ne from a flat jet of cold salty water. Highly super-specular scattering behavior dominates at each scattering geometry that is attributed to anisotropic momentum transfer to the surface. The TD channel exhibits a characteristic cos *θ* angular distribution and the TD fractions are similar to those reported for dodecane. Fitting the fractional energy loss in the IS channel to a soft-sphere kinematic model as a function of deflection angle shows that energy transfer to internal modes of the salty water surface is larger compared to the internal excitation of dodecane at similar beam energies.

## 2. Experimental methods

The scattering experiments are conducted with a crossed molecular beam apparatus^70,71^ modified for gas–liquid scattering as described previously.^59,62^ Briefly, the apparatus comprises three vacuum regions: a source chamber in which the molecular beam is created, a collision chamber where scattering with the liquid sample takes place, and a rotatable detector chamber housed within the collision chamber that contains an electron impact ionizer (80 eV electron kinetic energy), a quadrupole mass filter, and an ion detection assembly. The incidence angle *θ*_i_ of the beam with respect to the liquid surface normal is chosen to be 45°, 60°, or 75° by rotation of the chip holder. The scattering angle *θ*_f_ is also defined with respect to the surface normal, and the deflection angle follows as *χ* = 180° − (*θ*_i_ + *θ*_f_). The scattering angle is confined to a range between 90° − *θ*_i_ and 90° due to the geometry of the apparatus.

A pulsed supersonic (SS) molecular beam of 10% Ne seeded in He is produced by a piezoelectric valve (MassSpecpecD BV, Enschede).^72,73^ Stagnation conditions are typically 295 K and 3000 Torr, resulting in a beam velocity of 1572 ± 185 m s^−1^ (FWHM) with a mean Ne translational energy of 24.3 kJ mol^−1^. These values are derived from beam characterization *via* time-of-flight (TOF) measurements with a rotating (200 Hz) chopper wheel (two slits, 14 μs open time).

The cold salty water flat jet is formed in the collision chamber using a commercially available microfluidic chip.^58^ The liquid sample is initially prepared by vacuum-degassing 8 m LiBr dissolved in H_2_O (Milli-Q, MilliporeSigma) with pure He. This is done in a 2.3 L glass cylinder housed in a high-pressure stainless-steel reservoir. To deliver the solution with a flow rate of ∼2 mL min^−1^ and a temperature of ∼225 K at the interaction region, the reservoir is pressurized with pure helium to between 80 and 90 bar. A high-resolution camera captures calibrated images of the jet in operation, and typical dimensions of the first flat jet leaf are 0.4 × 1.2 mm^2^ (*W* × *H*). The thickness of the jet is not directly measured, but is estimated to be ∼1.5 μm at the center and thicker at the rims for pure water.^45^ These experimental conditions lead to a flow velocity of ∼7 m s^−1^. This velocity and the length of the jet leaf limit the detector viewing time to ∼150 μs. A 1.5 mm circular aperture is used at the detector entrance for all scattering experiments. Much of the collision chamber interior is covered with a liquid nitrogen-cooled copper wall that serves to both reduce the pressure inside the chamber by adsorbing gas particles on its surface and function as a heat sink for the liquid jet assembly through a connected Cu framework.

Numerous modifications to the liquid delivery setup were made since performing the dodecane experiments. The liquid passes through an in-line stainless-steel counter-current precooling stage ∼1 m in length held at 235 K by the use of a circulating LN_2_-cooled ethanol bath (Xylem Rule iL280P). In the collision chamber, the liquid is further cooled at the stainless-steel/marine-grade 464 brass chip holder to which flexible tinned Cu braids are attached. The braids are mounted to an extension of the cryogenically cooled Cu wall within the collision chamber and are designed such that they do not hinder rotation of the chip. The temperature of the chip holder *T*_holder_ is recorded by a thermocouple, and the temperature difference *T*_holder_ − *T*_liq_ (the true liquid temperature at the interaction region) is estimated to be less than 3 °C due to the relatively low vapor pressure of the solution at *T*_holder_.^47,49^ For Ne scattering at *θ*_i_ = 45°, 60°, and 75°, *T*_holder_ was measured to be 230 ± 1 K (*P*_vap_ = 7.6 × 10^−2^ Torr).

TOF measurements determine the translational energy distributions of scattered species at each scattering angle. Time zero is defined as the moment when the most intense part of the pulsed molecular beam reaches the interaction region. The temporal resolution of these measurements is then limited by the duration of the pulsed beam. For the Ne beam, the valve opening time is set to 15 μs, resulting in a measured temporal width at the detector of 29 μs. “Beam-off” data are subtracted from “beam-on” data, and TOF profiles are measured in a back-and-forth manner to achieve background-subtracted scattering spectra and thus intensity-calibrated angular distributions by calibrating to the *θ*_f_ = 90° spectra due to the high signal-to-noise ratio present at that angle. When generating the angular distributions, the first set of spectra collected when the detector is initially exposed to the scattered products is typically discarded due to substantial improvement in detector signal stability over time. Total acquisition times in the case of Ne scattering are typically between 20 and 40 min for a single spectrum.

## 3. Results and analysis

### 3.1. Scattering

**Fig. 1:**
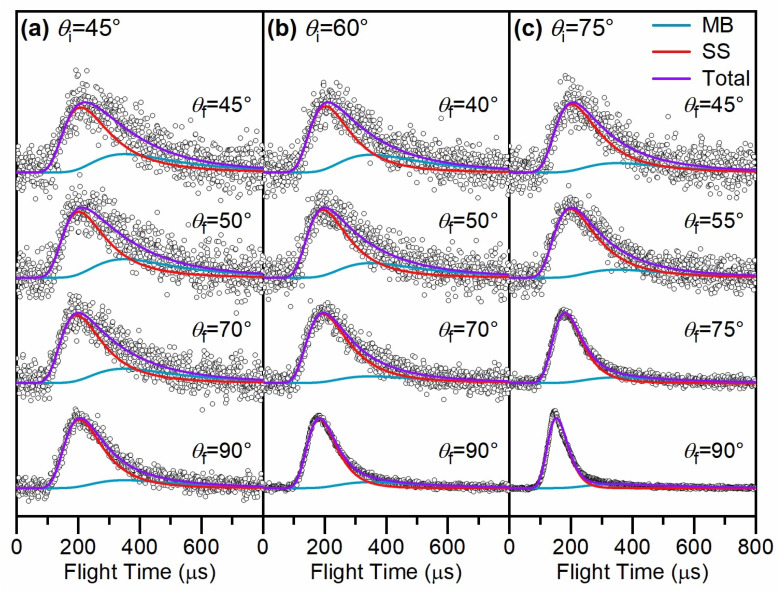
Normalized time-of-flight (TOF) spectra of Ne scattering from a cold salty water jet at (a) *θ*_i_ = 45°, (b) *θ*_i_ = 60°, and (c) *θ*_i_ = 75°. For *θ*_i_ = 60° and 75°, the scattering signal at *θ*_f_ = 90° is contaminated with “beam leakage” (see text). The data are fitted by the sum (purple traces) of a supersonic (SS) distribution (red traces) and a Maxwell–Boltzmann (MB) distribution (blue traces) at the liquid jet temperature. The mean translational energy *E*_i_ for Ne is 24.3 kJ mol^−1^.

**Fig. 2:**
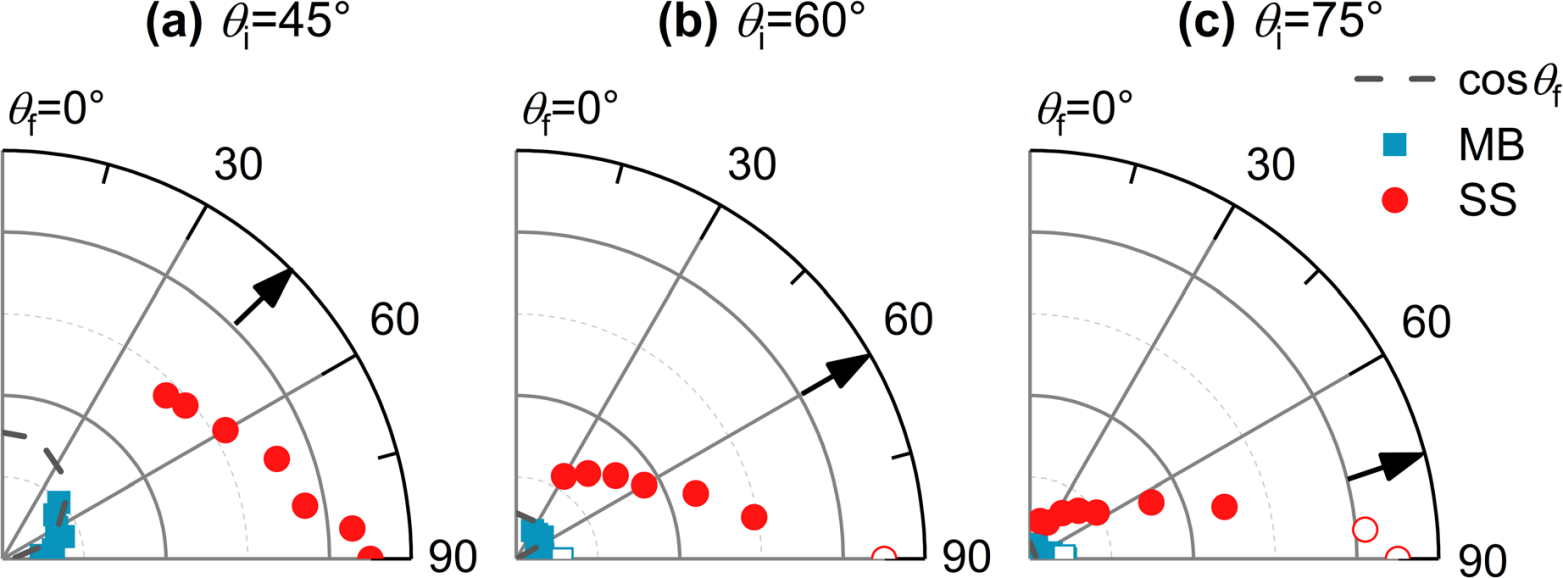
Angular plots created from the integrated, non-normalized intensities of Ne scattering at (a) *θ*_i_ = 45°, (b) *θ*_i_ = 60°, and (c) *θ*_i_ = 75°. Blue squares represent the thermal desorption (TD, Maxwell–Boltzmann [MB] distribution) and red circles the impulsive scattering (IS, supersonic [SS] distribution) contributions to the time-of-flight (TOF) fits. Open symbols denote angles at which the overall scattering signal is contaminated with beam leakage. The cosine function representing the expected angular distribution for evaporation is indicated by the dashed gray curve. Arrows indicate the specular angle.

TOF spectra of Ne scattered from a cold salty water jet are shown in Fig. 1a–c at incidence angles *θ*_i_ = 45°, 60°, and 75° for select detector angles. TOF spectra for other detector angles are shown in Fig. S1.† Angular distributions are shown in Fig. 2a–c for these three incidence angles and are derived from integrating the fitted TOF spectra. As *θ*_f_ increases, the TOF spectra shift toward earlier arrival times for all incidence angles. Additionally, the TOF spectra overall shift slightly toward earlier arrival times as *θ*_i_ increases across similar values of *θ*_f_.

The TOF profiles are fitted using two components assigned to a faster contribution from IS and a slower one from TD following our previous work on scattering from a dodecane jet.^62^ The IS mechanistic channel is modeled by a flux distribution for a supersonic (SS) molecular beam,^74,75^1
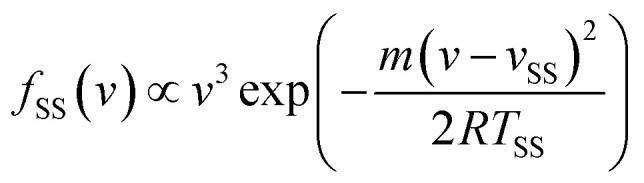
where *v* and *m* are the velocity and molecular mass of the particle in the scattered beam. *v*_SS_ and *T*_SS_ are the average flow velocity and temperature, respectively. The TD channel is modeled using a Maxwell–Boltzmann (MB) flux distribution,^66^2
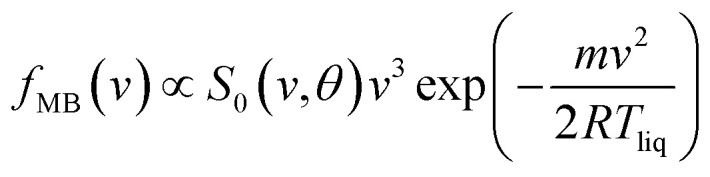
with *S*_0_(*v*,*θ*) being the velocity- and angle-dependent sticking coefficient and *R* the universal gas constant.

This fitting methodology is supported by measurements demonstrating Maxwellian behavior of Ar atoms evaporating from a salty water cylindrical jet at 237 K^76^ and was used to interpret our results on Ar evaporation from a cold salty water flat jet at 226 K, where near-Maxwellian behavior was observed at various values of *θ*_f_ with an overall cos *θ*_f_ angular distribution.^62^ Further, we assume a sticking coefficient of unity^66^ and limited interaction with the vapor sheath around the jet, consistent with scattering experiments that sample nascent scattering signal.

The scattering TOF spectra are fitted with a linear combination of SS and MB distributions describing the IS and TD scattering pathways, respectively.^65,77^ In the TD channel, *T*_liq_ is assumed to be equal to *T*_holder_, the value of which is reported in the previous section. Convolution with the molecular beam temporal profiles is carried out in the fitting procedure. The resulting best-fit SS and MB contributions are shown as red and blue traces, respectively, in Fig. 1a–c. These fits are also shown in Fig. S1† for other detector angles. Across all incidence angles *θ*_i_, the relative contribution from TD decreases as *θ*_f_ increases. This trend, and the narrowing of the TOF distributions as *θ*_f_ increases, reflect the cos *θ*_f_ angular distribution for TD. Additionally, as *θ*_i_ increases toward more grazing incoming trajectories for a given *θ*_f_, the IS/TD ratio increases, once again leading to narrower TOF profiles.

Fig. 2a–c shows the angular dependencies of the integrated IS and TD fits for *θ*_i_ = 45°, 60°, and 75°. The TD angular distributions are easier to discern in the zoomed-in angular plots shown in Fig. S2.† For all three incidence angles, the IS channel exhibits a strong deviation from the specular scattering expected for elastic collisions. Instead, super-specular scattering is observed, where the maximum intensity of the IS channel is at *θ*_f_ = 90°. However, for *θ*_i_ = 75°, where the effective target area of the flat jet is smallest, the TOF profiles for *θ*_f_ = 85° and 90° are contaminated from signal due to part of the molecular beam passing by the edge of the jet and subsequently entering the detector chamber; this “beam leakage” artificially enhances the signal near 90°. This is also observed for *θ*_i_ = 60° and *θ*_f_ = 90°.

Inspecting the angular dependence of the TD channel at *θ*_i_ = 45° shows that it follows a cos *θ*_f_ angular distribution, demonstrating that trapped Ne atoms undergo thermal equilibration with the liquid surface prior to desorption, akin to evaporation. Similar but less definitive results are observed for *θ*_i_ = 60° and 75° (see Fig. S2†). The cos *θ*_f_ angular distributions for TD imply that the vapor sheath around the jet does not significantly distort the nascent scattering signal. Overall, the increase in IS/TD ratios as both *θ*_i_ and *θ*_f_ increase is consistent with other gas–liquid scattering experiments.^62,78–80^

**Fig. 3:**
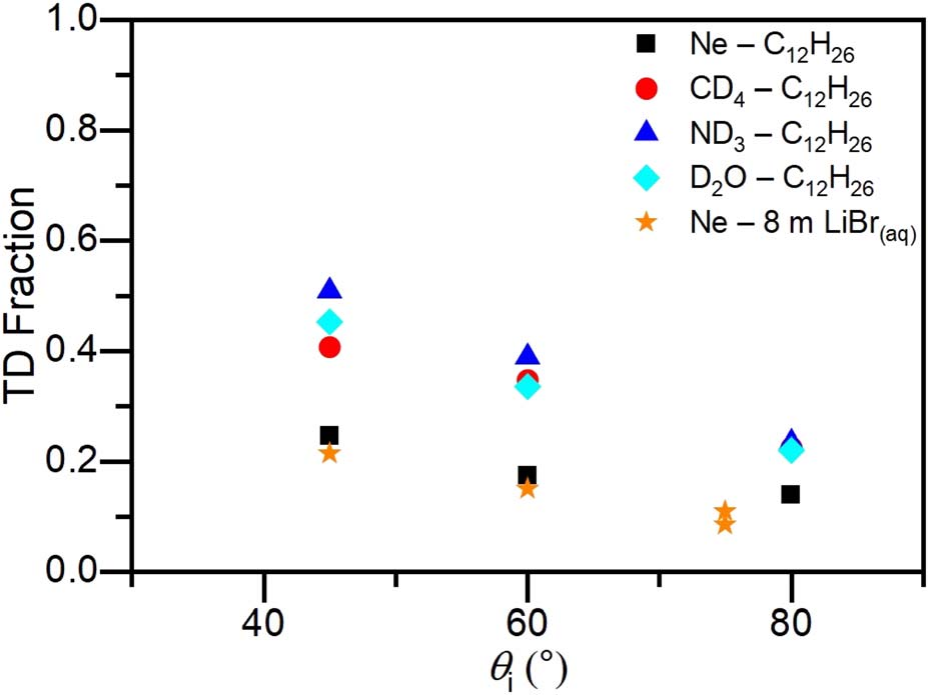
TD fraction at *θ*_f_ = 60° as a function of incidence angle *θ*_i_ for Ne scattered from cold salty water and dodecane flat jets, and CD_4_, ND_3_, and D_2_O scattered from a dodecane flat jet. For scattering from cold salty water at *θ*_i_ = 75°, TD fractions are plotted for *θ*_f_ = 55° and 65°. The dodecane data are taken from ref. 62.

**Fig. 4:**
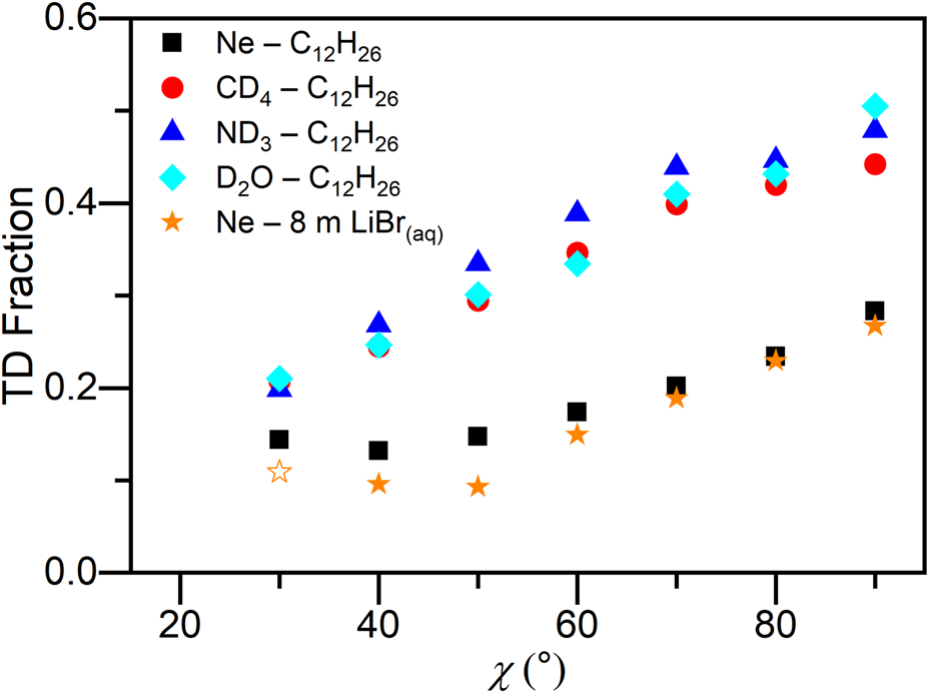
TD fraction as a function of deflection angle *χ* for Ne scattered from cold salty water and dodecane flat jets, and CD_4_, ND_3_, and D_2_O scattered from a dodecane flat jet at *θ*_i_ = 60°. Open symbols denote TD fraction values that are contaminated with beam leakage. The dodecane data are taken from ref. 62.

The TD fraction, defined as TD/(TD + IS) using the integrated intensities of each fitted curve, is plotted as a function of incidence angle *θ*_i_ and deflection angle *χ* for Ne scattering from cold salty water in Fig. 3 and 4, respectively. Our previous results scattering from dodecane^62^ are also shown for reference. As was reported for dodecane, the TD fraction decreases as *θ*_i_ increases. This was also observed for other gas–liquid scattering studies^78–80^ and differs from previous gas–solid experiments.^81–83^ For all three incidence angles, the TD fraction for Ne scattering from cold salty water is similar to that for Ne scattering from dodecane. This trend continues at each deflection angle for scattering at *θ*_i_ = 60° in Fig. 4. In addition, the TD fraction increases as *χ* increases for both systems.

### 3.2. Kinematic models

We analyze energy transfer at the gas–liquid interface in the IS channel using the fits to the scattering TOF profiles and the incident beam energy. A description of the average fractional energy loss in the IS channel as a function of deflection angle then follows according to the well-established^62,84,85^ “soft-sphere” kinematic model,^86,87^3
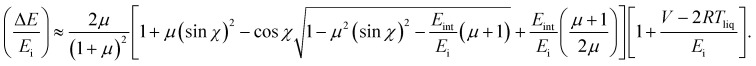
where the absolute change in translational energy for scattered particles is Δ*E* = *E*_i_ − 〈*E*_IS_〉, with incident translational energy *E*_i_ and average energy in the IS channel 〈*E*_IS_〉. Other parameters include the mass ratio *μ* = *m*_gas_/*m*_eff_ between the gas molecule and the effective surface mass, the deflection angle *χ* = 180° − (*θ*_i_ + *θ*_f_), the total internal excitation *E*_int_, and the gas–surface interaction potential *V*. Although this model does not report on the partitioning of *E*_int_ into the scatterer or surface, the internal excitation recovered for Ne (with no internal modes of its own) exclusively represents that of the surface.

**Fig. 5:**
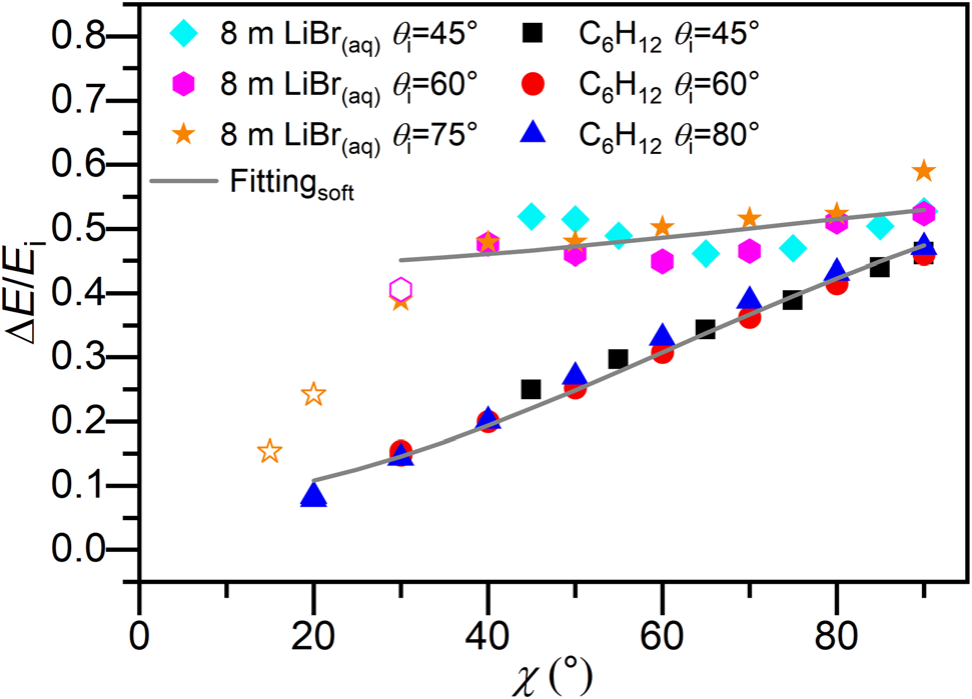
Average fractional energy loss as a function of deflection angle *χ* for impulsively scattered Ne from cold salty water and dodecane flat jets, with incident beam energies of 24.3 and 23.7 kJ mol^−1^, respectively. Open symbols denote fractional energy loss values that are contaminated with beam leakage. The solid curves give predictions for the soft-sphere model, where the incident particle interacts with a localized region of the surface with an effective mass, *m*_eff_, and this may increase its internal energy, *E*_int_, during a collision. The fitting results for Ne scattering from cold salty water and dodecane with the soft-sphere model are *m*_eff_ = 250 and 61 amu and *E*_int_ = 11.8 and 2.1 kJ mol^−1^, respectively. The dodecane data are taken from ref. 62.

Fig. 5 shows the measured fractional translational energy loss as a function of deflection angle for Ne scattering from both cold salty water and dodecane flat jets. As in Fig. 2, angles at which the fractional energy loss values are affected by contamination from beam leakage, and thus anomalously low, are denoted by open symbols and omitted from the soft-sphere model fitting procedure. As *χ* increases, Δ*E*/*E*_i_ also increases, and there is minimal dependence on the incidence angle. For *θ*_i_ = 60°, the energy loss values for Ne scattering from cold salty water increase from 0.48 to 0.52 between *χ* = 40° and 90°. Compared to Ne scattering from dodecane, with analogous values ranging from 0.20 to 0.46, the fractional energy loss values are higher overall but exhibit a weaker dependence on *χ*. This trend persists for the other two incidence angles.

The parametric fits from the soft-sphere model on the fractional energy loss data are also shown in Fig. 5, yielding values for *m*_eff_ and *E*_int_. The hard-sphere model fit, where *E*_int_ has been set to zero, is shown in Fig. S3.† As an approximation, the gas–surface interaction potential is estimated to be equivalent to the Lennard-Jones well depth between Ne and water, or 1.1 kJ mol^−1^.^88,89^ If the system is assumed to be noninteracting and *V* is set to zero, the fits do not change substantially. After implementing the soft-sphere model, *m*_eff_ is found to be 250 amu and *E*_int_ 11.8 kJ mol^−1^, compared to values of 61 amu and 2.1 kJ mol^−1^ for Ne scattering from dodecane. The high degree of correlation between *m*_eff_ and *E*_int_ complicates the reliable determination of their error bars; the procedure outlined in the SI yields estimated error bars of +100/−60 amu and ±1.6 kJ mol^−1^, respectively, for the two parameters.

The soft-sphere model provides an adequate fit to the fractional energy loss from cold salty water, although the fit was better for dodecane. However, the effective surface mass is much larger than the mass of a single water molecule or any ionic species that may be present at the surface. This result may indicate that collisions in the IS channel are more complex than simple two-body collisions, a scenario that goes beyond the soft-sphere model and indicates a potential breakdown of the model. We take care not to overinterpret this fitted value, since the soft-sphere model does not appropriately account for more complex scenarios such as multiple-bounce collisions. For such trajectories, an effective surface mass of 250 amu may not be representative of the kinematics at play. Further discussion on this point follows in the next section.

## 4. Discussion

Major findings in this work include super-specular scattering observed at all three incidence angles that were investigated, facile energy transfer in the IS channel, and the branching ratios between IS and TD mechanistic channels. We compare these results to our recent work on dodecane^62^ and work done by Saecker and Nathanson^90^ in which Ne was scattered from both squalane and glycerol surfaces at a fixed deflection angle of 90°.

In Fig. 2a–c, we observed a high degree of super-specular scattering at *θ*_i_ = 45°, 60°, and 75°, where the IS channel intensity reaches its maximum at an outgoing angle of 90°, albeit less sharply for 45° than for the other two incidence angles. This phenomenon has been seen to a considerably smaller extent in select gas–solid scattering systems^91,92^ and our prior scattering experiments from dodecane jets where the IS distribution peaked at an outgoing angle of 70° for *θ*_i_ = 60°.^62^ It contrasts with CO_2_ and OH scattering from a perfluorinated liquid surface in which sub-specular scattering was seen.^57,80^ Based on the gas–solid scattering literature, we attribute this effect to the anisotropic loss of momentum favored parallel to the surface normal.

The high degree of super-specular scattering seen here is consistent with the large fractional translational energy loss values, exceeding 40% for all deflection angles (Fig. 5), when compared to dodecane. Analyzing the fractional energy loss values for *θ*_i_ = 60° and assuming these values contribute to momentum transfer entirely along the surface normal (no momentum transfer parallel to the surface), we find that trajectories with Δ*E*/*E*_i_ ≥ 0.25 lead to a value of 90° for *θ*_f_. For a range of *χ* = 40° to 60° (the range over which most of the IS flux lies), the fractional energy loss values for cold salty water and dodecane are 0.48 to 0.45 and 0.20 to 0.31, respectively. These considerations imply that Ne scattering from cold salty water but not dodecane can be highly super-specular, in agreement with experiment.

While these kinematics offer a possible mechanism for the observed angular distribution, they do not explain this phenomenon, and a dynamical understanding of why momentum transfer to the surface is so anisotropic has yet to be formulated. In this regard, a possible microscopic mechanism lies in multiple-bounce collisions, which are enhanced for rough surfaces like liquids.^93^ MD simulations have shown that multiple-bounce collisions lead to a higher degree of energy loss,^68,69^ resulting in trajectories that are super-specular in nature.

The fractional translational energy loss in the IS channel plotted in Fig. 5 shows a higher degree of energy transfer to cold salty water than to dodecane at comparable beam energies. Overall, for both systems, fractional energy loss depends exclusively on *χ* and not independently on *θ*_i_ and *θ*_f_. The slight deviations in the *θ*_i_ = 45° data (where the IS and TD distributions temporally overlap to a greater degree) from the *θ*_i_ = 60° and 75° data at smaller values of *χ* is likely due to the conventional scattering mechanistic dichotomy failing to capture more nuanced scattering behavior such as multiple-bounce collisions, which have been shown to contribute to scattering signal for liquid surfaces.^68,93,94^

Comparing Ne scattering across the four different surfaces at hand, we find the trend for fractional energy loss in the IS channel at *χ* = 90° to be cold salty water (0.52) > dodecane (0.46) > squalane (0.42) > glycerol (0.38); note that the squalane and glycerol experiments were performed at *E*_i_ = 25 kJ mol^−1^.^90^ While results for squalane and glycerol are available only at *χ* = 90°, Fig. 5 shows that the deviation between water and dodecane results is considerably larger at smaller values of *χ*, *e.g.* 0.48 and 0.20 at *χ* = 40° for cold salty water and dodecane, respectively.

The fitted values for the internal excitation of the surface of *E*_int_ = 11.8 and 2.1 kJ mol^−1^ for cold salty water and dodecane, respectively, reveal that the cold salty water surface is internally excited to a greater degree. Without the presence of strong attractive forces, fractional energy loss (and the effective surface mass, which will be discussed later) has been previously used as a metric for determining the “softness” of a surface, or to what degree collisions with a surface are soft-spherelike.^90^ Here, *E*_int_ is a direct measure of surface softness, since *E*_int_ = 0 for a hard-sphere model. With this argument, we find that cold salty water is a softer surface than dodecane.

Contrasting with dodecane^62^ and squalane,^90^ the greater internal excitation energy of cold salty water suggests that the hydrogen-bonded network of liquid water allows for multiple low-frequency intermolecular modes to be excited by the incident Ne atoms. For example, THz spectroscopy of liquid water shows that there is a high density of intermolecular vibrational modes below 300 cm^−1^.^95^ Additionally, several librational modes of liquid water could be excited, including the twisting libration band at 485 cm^−1^ and rocking–wagging libration bands at 707 and 743 cm^−1^.^96^ Although the internal excitation is large compared to dodecane, the lowest lying vibrational mode for water, *ν*_2_ (1595 cm^−1^),^97^ is unlikely to be populated to a significant degree. Note that the hydrogen-bonded network, and thus these librational modes, are likely to be perturbed by the high ion concentration. Previous infrared spectroscopy data of highly concentrated (up to 12 M) LiBr salt solutions have shown shifts in the librational band maximum on the order of 100 cm^−1^ compared to pure water.^98^

The relative insensitivity of fractional energy loss with respect to *χ* is consistent with the large effective surface mass for cold salty water of 250 amu within the framework of the soft-sphere model.^84,86,87^ In prior work, large effective surface masses correlated with harder surfaces in the absence of attractive forces.^90^ This is not always the case, however. For example, Ar scattering at 80 kJ mol^−1^ from squalane and perfluoropolyether wetted wheels at 290 K led to effective surface mass and internal excitation values of 162 and 142 amu and 33.6 and 9.6 kJ mol^−1^, respectively.^78,84^ Within this context, the relatively large *m*_eff_ of 250 amu would suggest that collisions in the IS channel couple strongly with the hydrogen-bonded network present in the liquid. This counterintuitive result, where large energy transfer values are accompanied by a large *m*_eff_, was observed previously for oxygen and argon atomic scattering from liquid and self-assembled monolayer surfaces, referenced above, and attributed to the number of surface atoms participating in a collision event.^84,93^ However, such an interpretation must be considered with caution in light of the possible breakdown of the soft-sphere model discussed at the end of Section 3.

The relatively large *m*_eff_ may also suggest the presence of ions, specifically bromide anions, at the interface. Although recent vibrational sum-frequency generation work has shown that anions are not enhanced at the interface of salty water solutions, larger halide anions were found to have a higher propensity to concentrate at the interface.^99^ Further, this study was confined to lower concentrations of salt (≤1.0 M) and NaBr, not LiBr, for investigating bromide behavior. The relatively high concentration of Br^−^ in our solution, together with the large effective surface mass and fractional energy loss values, make it plausible that Ne interacts with Br^−^ at the interface. Further studies must be conducted in tandem with MD simulations on highly concentrated salty water solutions to determine the degree of anion enhancement at the interface.

The similar TD fractions for cold salty water compared to dodecane suggest that, surprisingly, cold salty water is a similarly accommodating surface as dodecane with respect to incident Ne atoms.^90^ Moreover, the TD fractions for Ne scattering from the two liquids are notably less than those for CD_4_, ND_3_, and D_2_O from dodecane (Fig. 4). While scattering from cold salty water results in larger energy transfer values compared to scattering from dodecane, our findings suggest that energy transfer and TD fraction reflect two different aspects of the gas–solvent interaction. In particular, Nathanson has proposed that the TD fraction correlates with the free energy of solvation for the scatterer in the solvent,^90^ whereas energy transfer in the IS channel more likely reflects interfacial dynamics. To our knowledge, there are no reliable free energy of solvation values for Ne in cold salty water, so it remains to be seen whether this correlation holds across the two liquids under the rather different conditions of the two experiments (*T*_liq_ = 230 K for cold salty water *vs.* 269 K for dodecane). However, recent results in our group show that there is essentially no IS channel for ND_3_ scattering from cold salty water, a result consistent with the high solubility of ND_3_ in water, while the TD fraction for CD_4_ scattering is similar to Ne.^100^ It thus seems reasonable that the TD fraction for scattering from cold salty water is in fact strongly correlated with solubility.

Examining the TD fractions plotted as a function of incidence angle in Fig. 3, more grazing collisions (*i.e.*, larger *θ*_i_) result in less TD, consistent with other gas–liquid scattering experiments.^78–80^ In gas–solid scattering,^81–83,86^ the opposite trend is observed. The relationship between normal energy scaling and the likelihood of trapping at the interface does not hold for liquid surfaces, presumably due to the increased roughness and corrugation which converts incident kinetic energy in large *θ*_i_ collisions into translational motion away from the surface.^80^

## 5. Conclusions

Herein, we have conducted the first angle-resolved scattering measurements of Ne from a cold salty water flat jet. This expands upon our previous work scattering from a dodecane flat jet and demonstrates advancement of the technique to probe dynamics that occur at volatile liquid surfaces. We have also elucidated mechanistic branching ratios and energy transfer across a wide range of deflection angles at the vapor–water interface. Key results include the super-specular angular distributions derived from the TOF profiles, the related energy transfer in the IS channel, and similar TD fractions between cold salty water and dodecane.

The angular distributions for Ne scattering from cold salty water at *θ*_i_ = 45°, 60°, and 75° all exhibit an approximately cos *θ*_f_ distribution in the TD channel and highly super-specular scattering in the IS channel, contrasting with our previous work scattering from dodecane jets. The former observation validates the presence of nascent scattering conditions, while the latter is attributed to anisotropic momentum transfer along the surface normal, mediated by the high degree of fractional energy loss in the IS channel.

Fitting the measured fractional energy loss values as a function of deflection angle with a soft-sphere kinematic model yields internal excitation and effective surface mass values. The relatively large internal excitation value of 11.8 kJ mol^−1^ for cold salty water shows that it is a softer surface than dodecane. The amount of internal excitation present can populate multiple collective intermolecular modes of the hydrogen-bonded network but not discrete vibrational modes of single water molecules.

The large effective surface mass of 250 amu is consistent with the relatively small dependence of fractional energy loss values on *χ* for cold salty water and suggests that IS trajectories couple strongly with the hydrogen-bonded network present in the liquid. It may also indicate the presence of bromide ions at the surface of cold salty water. However, as discussed above, the possible contribution of multiple-collision trajectories to the IS channel complicates the interpretation of *m*_eff_. The TD fractions for Ne scattering from cold salty water are similar to those for dodecane, indicating somewhat surprisingly that cold salty water is a similarly accommodating surface as dodecane.

The scattering results presented here provide a fundamental understanding of scattering dynamics at the vapor–water interface and continue to show the unique ability of the flat liquid jet technique in uncovering interfacial gas–liquid interactions. This work will serve as the foundation for future angle-resolved scattering studies on aqueous systems. Studies on nonreactive and reactive scattering from cold salty water flat jets, including polyatomic scattering, are currently underway, and experiments to investigate the effect of surfactants on scattering dynamics are in development.

## Data availability

The data that support the findings of this study are available from the corresponding author upon reasonable request.

## Author contributions

Walt Yang: conceptualization (equal); data curation (lead); formal analysis (lead); investigation (lead); methodology (equal); software (equal); writing – original draft (lead); writing – review & editing (equal). Madison M. Foreman: conceptualization (equal); data curation (equal); formal analysis (equal); investigation (equal); methodology (equal); software (supporting); writing – review & editing (equal). Tiffany C. Ly: data curation (supporting); formal analysis (supporting); investigation (supporting); methodology (supporting); software (supporting); writing – review & editing (supporting). Kevin R. Wilson: conceptualization (supporting); supervision (supporting). Daniel M. Neumark: conceptualization (lead); methodology (equal); project administration (lead); resources (lead); supervision (lead); validation (lead); writing – review & editing (equal).

## Conflicts of interest

The authors declare no conflicts of interest.

## Supplementary Material

SC-OLF-D5SC01636C-s001

SC-OLF-D5SC01636C-s002

SC-OLF-D5SC01636C-s003

SC-OLF-D5SC01636C-s004

SC-OLF-D5SC01636C-s005

SC-OLF-D5SC01636C-s006

SC-OLF-D5SC01636C-s007
