# Supplementary figures and images for: Molecular beam scattering of neon from flat jets of cold salty water
Source: Chem Sci. 2025 May 30;16(25):11608–18. doi: 10.1039/d5sc01636c (PMC12123539; doi:10.1039/d5sc01636c)

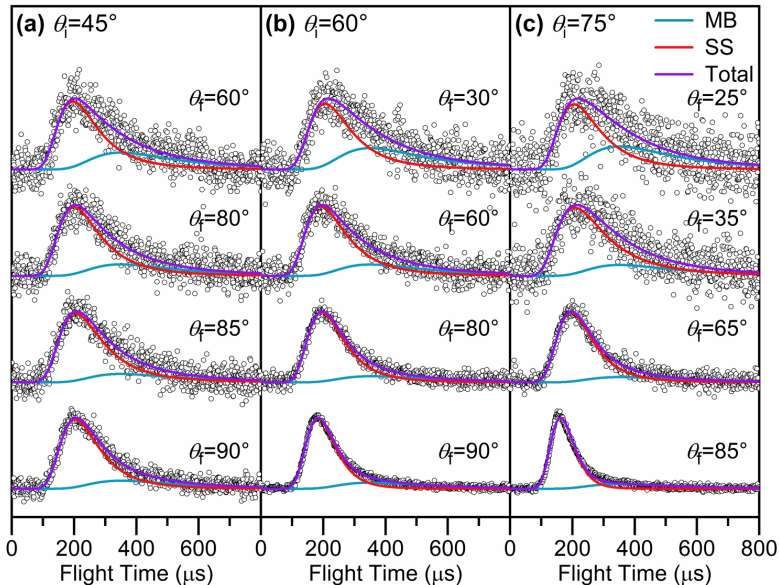

Supplement: SC-016-D5SC01636C-s002 [file SC-016-D5SC01636C-s002.pdf]

**(a)**  $\theta_i = 45^\circ$

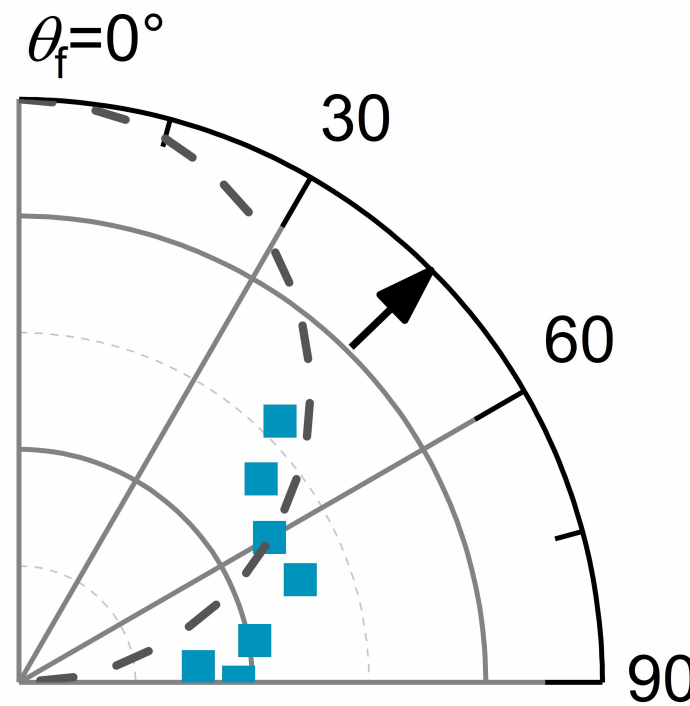

**(b)**  $\theta_i = 60^\circ$

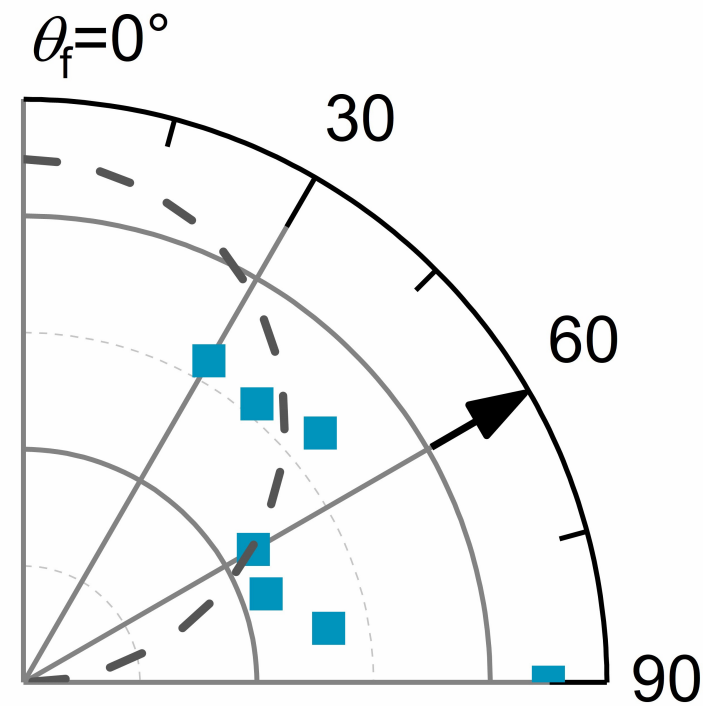

**(c)**  $\theta_i = 75^\circ$

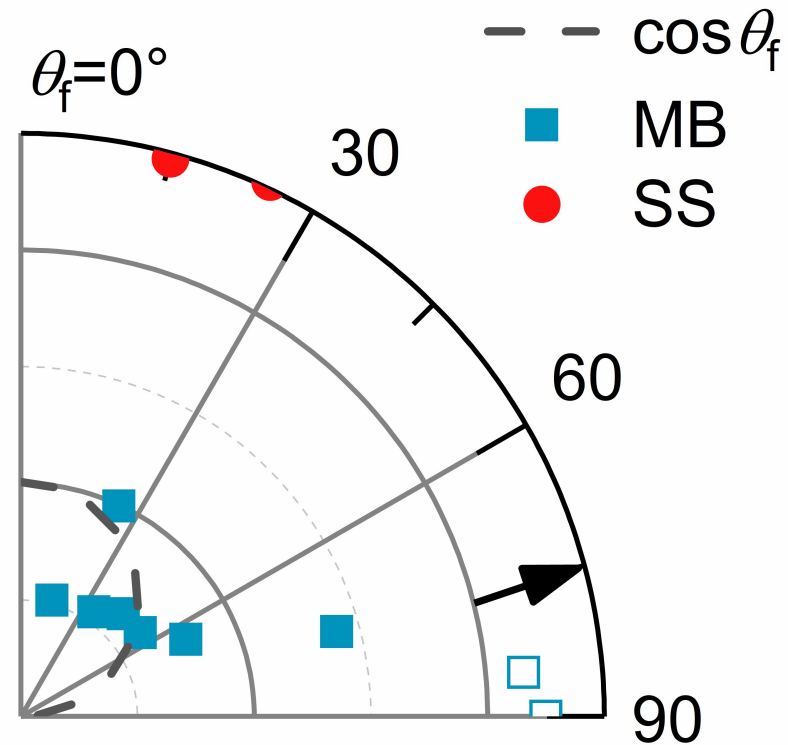

Supplement: SC-016-D5SC01636C-s003 [file SC-016-D5SC01636C-s003.pdf]

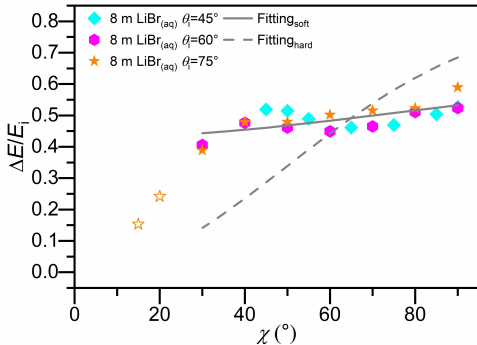

Supplement: SC-016-D5SC01636C-s004 [file SC-016-D5SC01636C-s004.pdf]

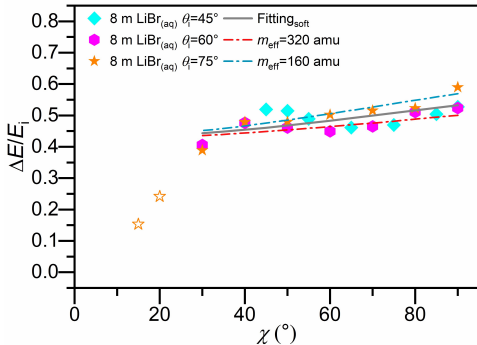

Supplement: SC-016-D5SC01636C-s005 [file SC-016-D5SC01636C-s005.pdf]

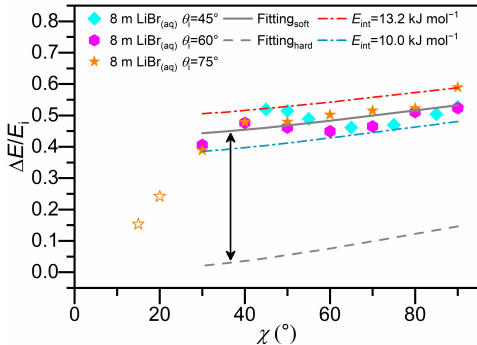

Supplement: SC-016-D5SC01636C-s006 [file SC-016-D5SC01636C-s006.pdf]
