# Supplementary material for: Molecular beam scattering of neon from flat jets of cold salty water
Source: Chem Sci. 2025 May 30;16(25):11608–18. doi: 10.1039/d5sc01636c (PMC12123539; doi:10.1039/d5sc01636c)
Supplement: SC-016-D5SC01636C-s007 [file SC-016-D5SC01636C-s007.pdf]

Supplementary Information (SI) for Chemical Science.
This journal is © The Royal Society of Chemistry 2025

Supporting Information

Molecular Beam Scattering of Neon from Flat Jets of Cold Salty Water

Walt Yang[1,2], *Madison M. Foreman*[1,2], *Tiffany C. Ly*[1], *Kevin R. Wilson*[2], *Daniel M. Neumark*[1,2,a]

[1] *Department of Chemistry, University of California, Berkeley, CA 94720, USA*

[2] *Chemical Sciences Division, Lawrence Berkeley National Laboratory, Berkeley, CA 94720, USA*

[a)] **Author to whom correspondence should be addressed: dneumark@berkeley.edu**

S1. Ne Scattering Time-of-Flight Spectra and Angular Distributions

Figure S1a–c shows TOF spectra of Ne scattered from a cold salty water flat jet at incidence angles θ_i = 45°, 60°, and 75° for detector angles that are not shown in the main text. The resulting best-fit SS and MB contributions, describing IS and TD, are shown as red and blue traces, respectively. Upon integrating the fits, the zoomed-in angular dependency of the TD pathway is shown in Figure S2a–c alongside a dashed gray curve representing $\cos\theta_f$.

S2. Hard-Sphere Kinematic Model Fitting

Figure S3 shows the parametric fits from both the soft-sphere and hard-sphere kinematic models on the fractional energy loss data, yielding values for m_{eff} and E_{int}. In the hard-sphere model, E_{int} has been set to zero. The soft-sphere model fitting results in m_{eff} = 250 amu and E_{int} = 11.8 kJ mol^{-1}, whereas the hard-sphere model predicts m_{eff} = 30 amu. While the value for m_{eff} from the hard sphere model might appear more reasonable, the fit to the data is clearly far worse.

S3. Error Analysis of Soft-Sphere Kinematic Model Fitting

It is necessary to comment on the degree of correlation m_{eff} and E_{int} have with one another in the soft-sphere model. The covariance matrix of the parameters fitted in the soft-sphere kinematic model[1-3] (Eq. 3) reports on the joint variability of, or the degree of correlation between $\mu = m_{gas}/m_{eff}$ and E_{int}. The one-standard deviation (1-SD) errors on these fitted parameters can be computed from the square root of the variances after diagonalizing the covariance matrix.[4] It can be shown that there exists a large area in the (μ, E_{int}) parameter space that share similar 1-SD errors in μ compared to the m_{eff} = 250 amu and E_{int} = 11.8 kJ mol^{-1} best-fit values in the main text. This is also the case for the 1-SD errors in E_{int}. Note that the 1-SD errors in E_{int} are many orders of magnitude larger than the typically fitted values for E_{int} in the soft-sphere model. Although it seems that the fitting is thus quite flexible, attempting to fit the Ne fractional energy loss data to other pairs of m_{eff} and E_{int} values leads to visually poorer fits. As a result, we have determined that the 1-SD errors, derived from the covariance matrix of the parameters, reports mainly on the degree of correlation m_{eff} and E_{int} have, and thus do not reliably represent the fitting error bars in the context of the soft-sphere fitting done here.

Instead, we estimate the error bars associated with the fitted m_{eff} and E_{int} values. Upon fixing E_{int} to be 11.8 kJ mol^{-1} and choosing values for m_{eff} of 350 and 190 amu, the red and blue dash-dotted curves result in Figure S4, respectively. Choosing these values of m_{eff} leads to distinguishably poorer fits in terms of the curvature of the predicted soft-sphere model, and thus we estimate the effective surface mass to be 250^{+100}_{-60} amu. The χ^2 values for the m_{eff} = 250, 350, and 190 amu fits are 0.039, 0.053, and 0.052, respectively. Such an estimation has been carried out in previous work.[3] For E_{int}, we show predictions for both the soft-sphere and hard-sphere (where E_{int} is assumed to be zero) models with m_{eff} = 250 amu in Figure S5 as solid and dashed gray curves, respectively. Due to the functional form of the model, E_{int} mainly encodes a fractional energy offset between the soft- and hard-sphere pictures,[3] which is represented by the black double-sided arrow. Following this, by fixing m_{eff} to 250 amu and choosing values for E_{int} of 13.4 and 10.2 kJ mol^{-1}, the red and blue dash-dotted curves in Fig. S4 appropriately bound the fractional energy loss values. Thus, we estimate the internal excitation to be 11.8 ± 1.6 kJ mol^{-1}. The χ^2 values for the E_{int} = 11.8, 13.4 and 10.2 kJ mol^{-1} fits are 0.039, 0.153, and 0.192, respectively.

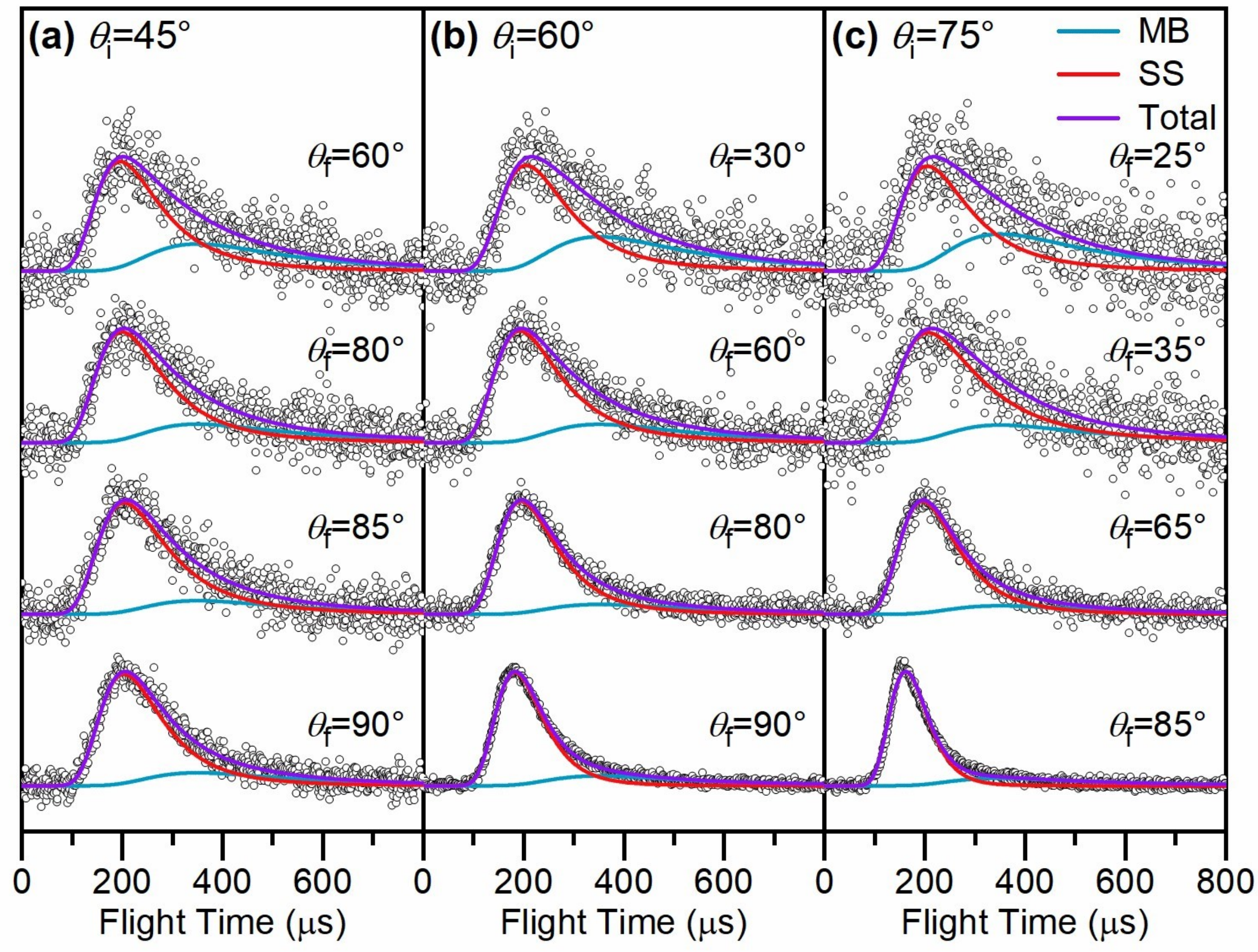

Figure S1. Normalized time-of-flight (TOF) spectra of Ne scattering from a cold salty water jet at (a) $\theta_i = 45°$, (b) $\theta_i = 60°$, and (c) $\theta_i = 75°$. For $\theta_i = 75°$, the scattering signal at $\theta_f = 85°$ is contaminated with "beam leakage" (see main text). The data are fitted by the sum (purple traces) of a supersonic (SS) distribution (red traces) and a Maxwell–Boltzmann (MB) distribution (blue traces) at the liquid jet temperature. The mean translational energy E_i for Ne is 24.3 kJ mol^{-1}.

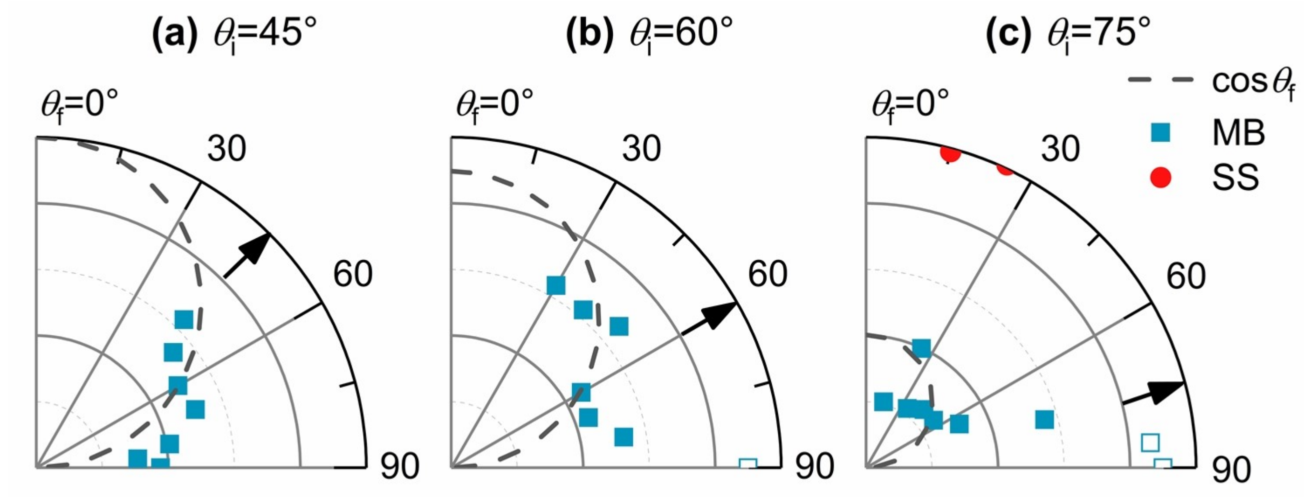

Figure S2. Zoomed-in angular plots created from the integrated, non-normalized intensities of Ne scattering at (a) θ_i = 45°, (b) θ_i = 60°, and (c) θ_i = 75°. Blue squares represent the thermal desorption (TD, Maxwell–Boltzmann [MB] distribution) and red circles the impulsive scattering (IS, supersonic [SS] distribution) contributions to the time-of-flight (TOF) fits. Open symbols denote angles at which the overall scattering signal is contaminated with beam leakage. The cosine function representing the expected angular distribution for evaporation is indicated by the dashed gray curve. Arrows indicate the specular angle.

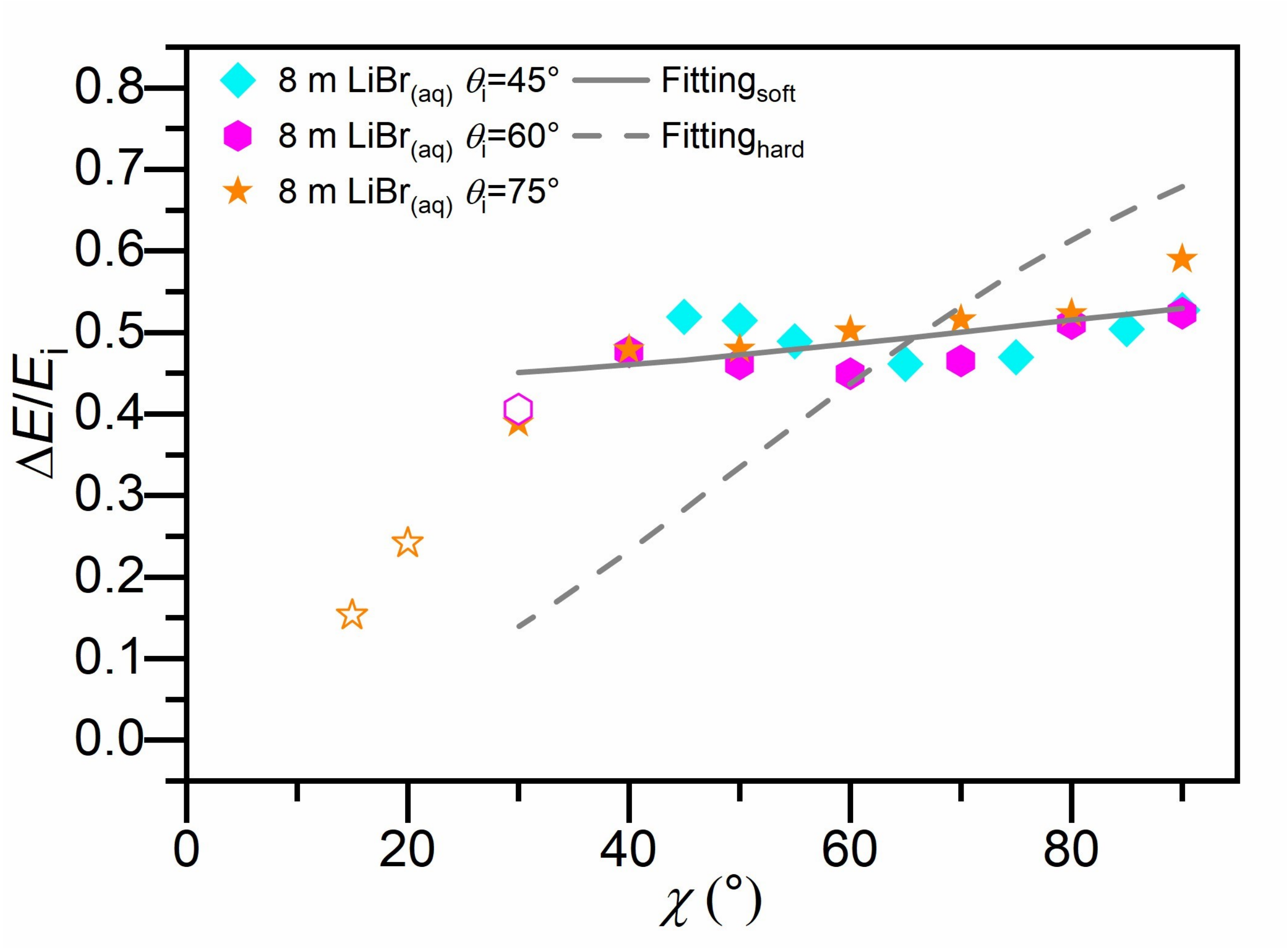

Figure S3. Average fractional energy loss as a function of deflection angle χ for impulsively scattered Ne from a cold salty water flat jet, with an incident beam energy of 24.3 kJ mol^{-1}. Open symbols denote fractional energy loss values that are contaminated with beam leakage. The solid curve gives the prediction for the soft-sphere model, where the incident particle interacts with a localized region of the surface with an effective mass, m_{eff}, and this may increase its internal energy, E_{int}, during a collision. The dashed curve gives the prediction for the hard-sphere model where E_{int} has been set to zero. The fitting result for Ne scattering with the soft-sphere model is m_{eff} = 250 amu and E_{int} = 11.8 kJ mol^{-1}, whereas the hard-sphere model predicts m_{eff} = 30 amu.

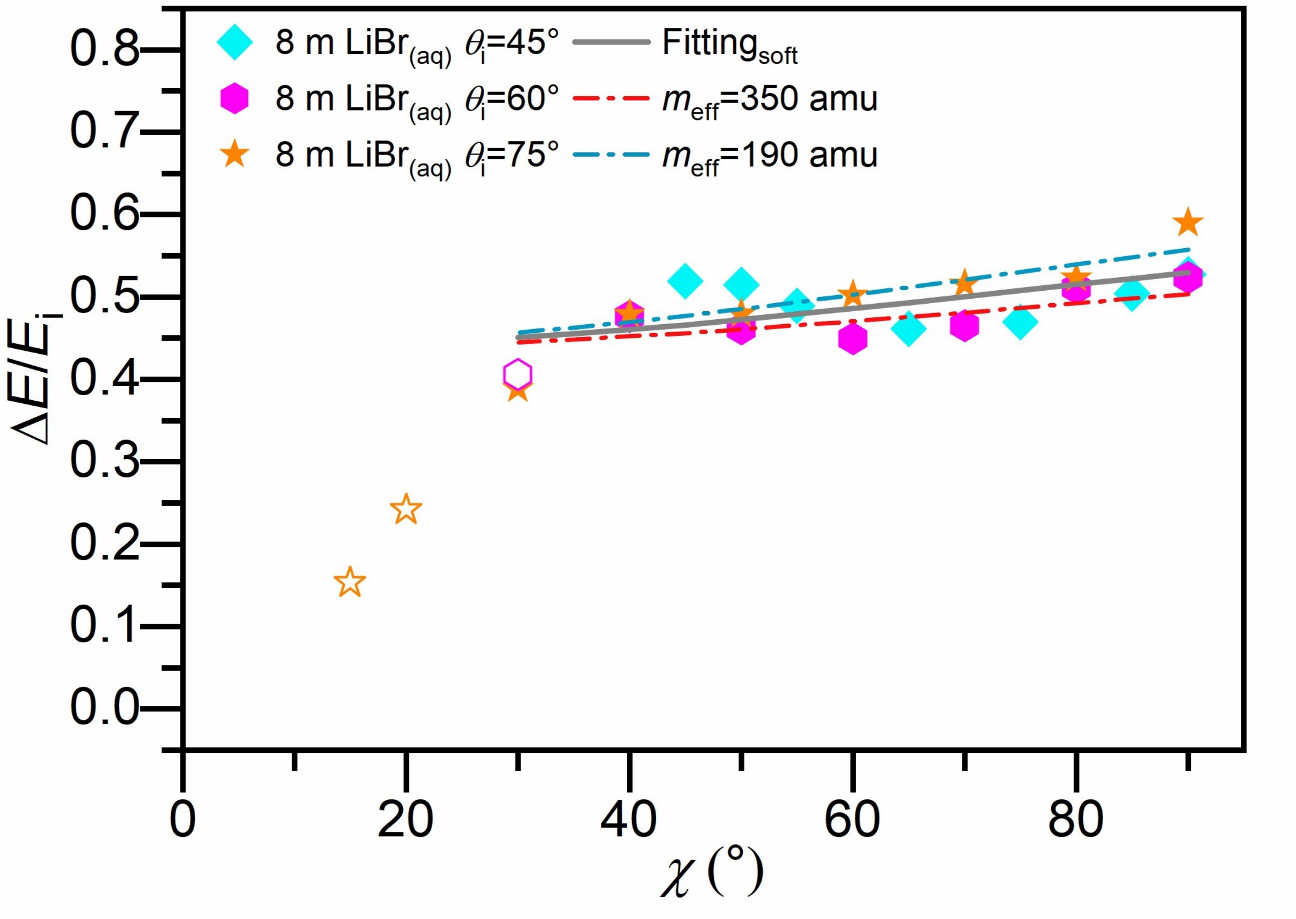

Figure S4. Average fractional energy loss as a function of deflection angle χ for impulsively scattered Ne from a cold salty water flat jet, with an incident beam energy of 24.3 kJ mol^{-1}. Open symbols denote fractional energy loss values that are contaminated with beam leakage. The solid curve gives the prediction for the soft-sphere model, where the incident particle interacts with a localized region of the surface with an effective mass, m_{eff}, and this may increase its internal energy, E_{int}, during a collision. The fitting result for Ne scattering with the soft-sphere model is m_{eff} = 250 amu and E_{int} = 11.8 kJ mol^{-1}. The red and blue dash-dotted curves give predictions for the soft-sphere model where E_{int} is constrained to 11.8 kJ mol^{-1} and m_{eff} = 350 and 190 amu, respectively.

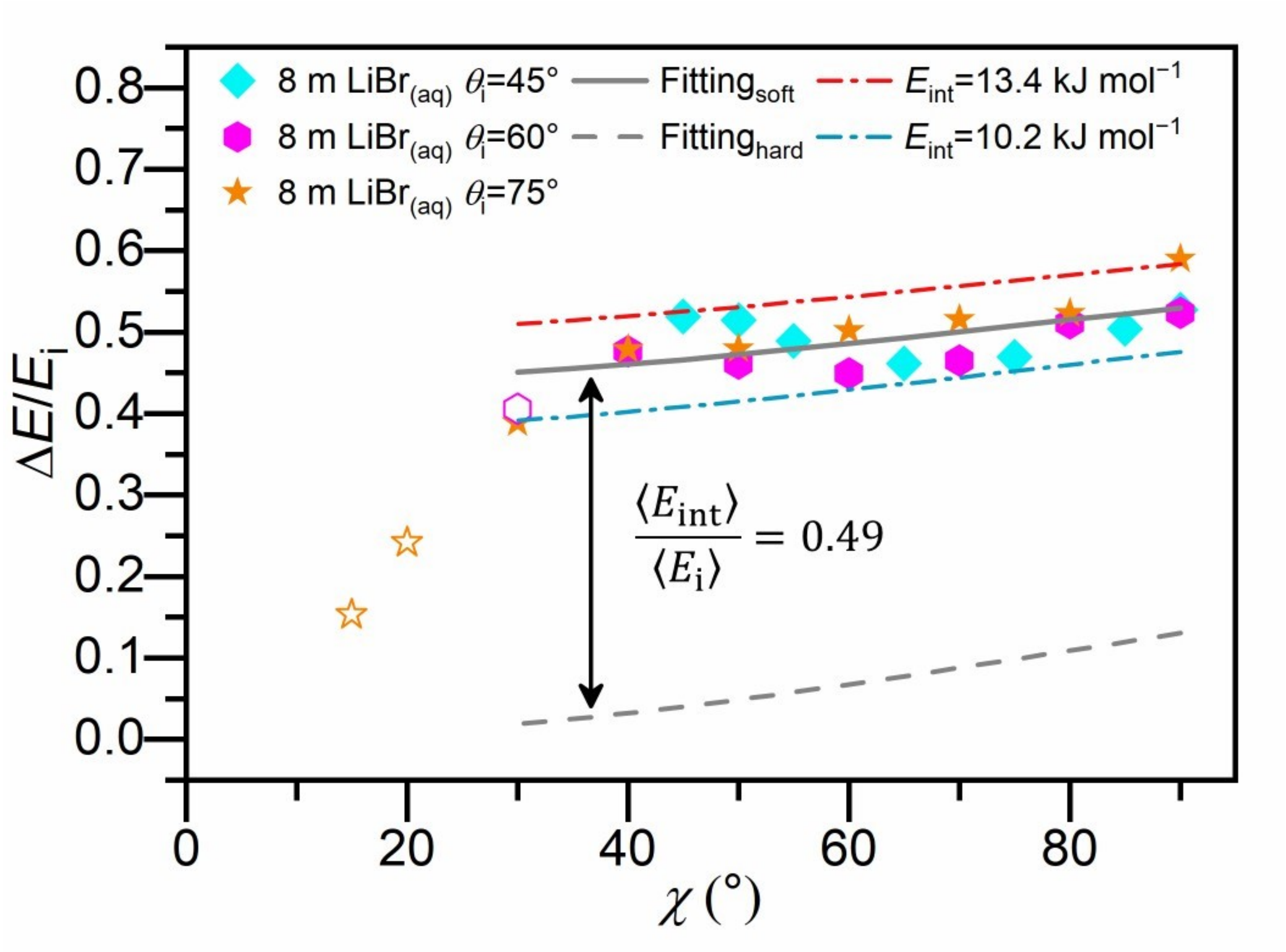

Figure S5. Average fractional energy loss as a function of deflection angle χ for impulsively scattered Ne from a cold salty water flat jet, with an incident beam energy of 24.3 kJ mol^{-1}. Open symbols denote fractional energy loss values that are contaminated with beam leakage. The solid curve gives the prediction for the soft-sphere model, where the incident particle interacts with a localized region of the surface with an effective mass, m_{eff}, and this may increase its internal energy, E_{int}, during a collision. The fitting result for Ne scattering with the soft-sphere model is $m_{\mathrm{eff}} = 250$ amu and $E_{\mathrm{int}} = 11.8$ kJ mol^{-1}. The dashed gray curve gives the prediction for the hard-sphere model with same m_{eff} as the soft-sphere model and where E_{int} is assumed to be zero. The red and blue dash-dotted curves give predictions for the soft-sphere model where m_{eff} is constrained to 250 amu and $E_{\mathrm{int}} = 13.4$ and 10.2 kJ mol^{-1}, respectively. The black double-sided arrow indicates the fractional energy offset between the soft- and hard-sphere models.

REFERENCES

(1) C. T. Rettner and M. N. R. Ashfold. In *Dynamics of Gas-Surface Interactions*, Royal Society of Chemistry, 1991.
(2) G. M. Nathanson, Molecular Beam Studies of Gas–Liquid Interfaces, *Annu. Rev. Phys. Chem.*, 2004, **55**, 231–255.
(3) W. A. Alexander, J. Zhang, V. J. Murray, G. M. Nathanson and T. K. Minton, Kinematics and dynamics of atomic-beam scattering on liquid and self-assembled monolayer surfaces, *Faraday Discuss.*, 2012, **157**, 355.
(4) K. W. Vugrin, L. P. Swiler, R. M. Roberts, N. J. Stucky-Mack and S. P. Sullivan, Confidence region estimation techniques for nonlinear regression in groundwater flow: Three case studies, *Water Resources Research*, 2007, **43**.
